# A web server for interactive and zoomable Chaos Game Representation images

**DOI:** 10.1186/1751-0473-4-6

**Published:** 2009-09-17

**Authors:** Kazuharu Arakawa, Kazuki Oshita, Masaru Tomita

**Affiliations:** 1 Institute for Advanced Biosciences, Keio University, Fujisawa, 252-8520, Japan

## Abstract

Chaos Game Representation (CGR) is a generalized scale-independent Markov transition table, which is useful for the visualization and comparative study of genomic signature, or for the study of characteristic sequence motifs. However, in order to fully utilize the scale-independent properties of CGR, it should be accessible through scale-independent user interface instead of static images. Here we describe a web server and Perl library for generating zoomable CGR images utilizing Google Maps API, which is also easily searchable for specific motifs. The web server is freely accessible at , and the Perl library as well as the source code is distributed with the G-language Genome Analysis Environment under GNU General Public License.

## Background

Genomic sequences exhibit characteristic nucleotide compositional bias, especially in the relative abundances of short oligonucleotides. While diverse dinucleotide frequencies are observed among various phyla, closely related species tend to display similar compositions [1]. Through these studies, the relative abundances of dinucleotides are considered to be the "genomic signature" [2,3]. Chaos Game Representation (CGR) was first proposed by Jeffrey as a scale-independent means to visualize this non-randomness of genomic sequences, by applying the concept of chaotic dynamical systems [4]. Further studies by Almeida *et al*. has shown that CGR is a generalized Markov chain probability table which can accommodate non-integer orders, and that CGR is advantageous over Markov transition tables for its computational efficiency and scale-independence [5-8].

Several software tools, including a database of CGR images [9], a web server [5], and a tool in the EMBOSS package [10], are already available for CGR analysis; however, these tools produce static images, which limits the full utility of CGR as scale-independent Markov transition table. Zoomable User Interface (ZUI) is effective in representing such scalable information [11], as exemplified by the popularity of Google Maps [12] in representing the geographical data. Therefore, here we describe a web server for generating interactive and zoomable CGR images, using Google Maps API [13] and Web 2.0 technologies [14].

## Implementation

### Chaos Game Representation

**Figure 1:**
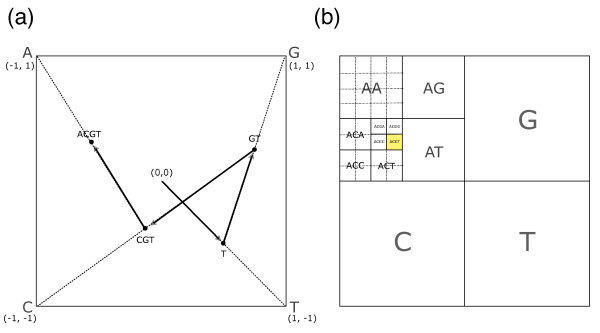
**Algorithm for CGR and *k*-mer table**. (a) CGR is generated by iterating the movement of pointer to the midpoint of current position and the vertex corresponding to the next nucleotide within a given sequence in reverse order. Here the reverse order is used to match the coordinate system of CGR and *k*-mer table. (b) *k*-mer table is generated by repeated division of the square into four quadrants representing the four nucleotides. Note that ACGT position is identical in CGR and *k*-mer table.

In order to generate CGR for a given nucleotide sequence, a rectangular coordinate system is defined to be confined by four vertices representing the four nucleotides: A (-1, 1), C (-1, -1), G (1, 1), and T (1, -1). Then, starting from the origin (0, 0), a pointer is moved to the midpoint of current position and the vertex corresponding to the next nucleotide. For example, for a tetramer sequence "ACGT", the pointer is first moved to the midpoint between the origin (0, 0) and T (1, -1), which is (0.5, -0.5), and then to the next midpoint between this position and G (1, 1), as shown in Figure 1a. Repeating this procedure for all nucleotide within the given sequence results in the CGR image. Note that by tracing the nucleotide in the reverse order, a given position within the CGR image contains the information about its trajectory, and thus retains the information about the Markovian relationships of successive nucleotides.

Mathematically, CGR is an iterated function system (IFS). For a given nucleotide sequence in reverse order *g *of length *n*_*G *_(*g*_*i *_∈ A, T, G, C} and *i *= 1,..., *n*_*G*_), CGR *X *(set of coordinates *X*_*i *_for *i *= 1,..., *n*_*G*_) is given by iterating the following function,

(1)

where *X*_*o *_= (0, 0), *V*(A) = (-1, 1), *V*(C) = (-1, -1), *V*(G) = (1, 1), *V*(T) = (1, -1).

### *k*-mer table

To generate *k-*mer table (or FCGR: frequency matrices extracted from CGR, as defined by Almeida *et al*. [5]), a square is repeatedly subdivided into four squares, while retaining the quadrant representation of four nucleotides, where A is upper left, C is lower left, G is upper right, and T is lower right. For example, for the tetramer "ACGT", upper left square representing A is subdivided, and then lower left square within this upper left square representing "AC" is subdivided, and so on (see Figure 1b for details). Repeating this process for all *k*-mer while color-coding the pixels with the abundance of corresponding *k*-mer from white (rare) to black (frequent) results in the *k*-mer table with 4^k ^pixels of width 2^k^.

Mathematically, the position of a certain oligomer can be calculated by converting the nucleotide sequence into two binary bit sequences corresponding to the horizontal and vertical coordinates. C and T bases move to the lower quadrants, and G and T bases move to the right quadrants. Therefore, by substituting A and G with 0 and C and T with 1, a binary number corresponding to the y-distance from top-left corner pixel of the image is obtained. Similarly, x-distance is obtained by substituting A and C with 0 and G and T with 1. For example, distance of ACGT from the upper-left corner pixel is given by (0011, 0101) in binary, which is (3, 5) in decimal. Therefore, ACGT is located at the 4th column, 6th row of the 16 × 16 pixel image (Figure 1b).

**Figure 2:**
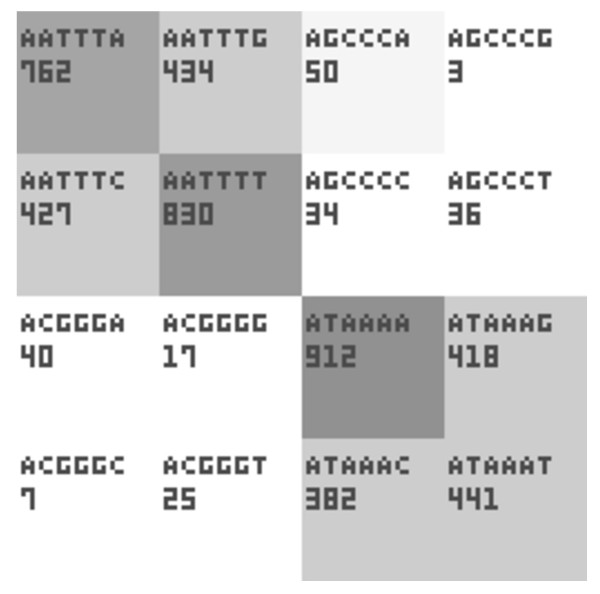
**Close-up of oligomer boxes in *k*-mer table**. In order to minimize the image size, 3 × 4 pixel font and 3 × 5 pixel font are used to represent the oligomer sequence and count, respectively. 32 × 32 pixel box can fit up to octamers.

Each box representing certain *k*-mer produced by our software shows the sequence and the count of *k*-mer (Figure 2). In order to minimize the file size, we have defined 3 × 4 pixel font for the nucleotide sequence and 3 × 5 pixel font for numbers.

### Web interface

The web server interface was developed with G-language Genome Analysis Environment version 1.8.9 [15-17], and Google Maps API [13] was used for ZUI. CGR and *k*-mer table are drawn using the GD library, and subsequently converted into Google Maps format using the *generateGMap *function of G-language GAE, which is computationally optimized by utilizing multiple CPU cores. Incremental search of composite oligomers was implemented with Google Maps API and Javascript. Resulting web server is available through the G-language REST server [18].

## Results and Discussion

### Interactive and zoomable user interface

**Figure 3:**
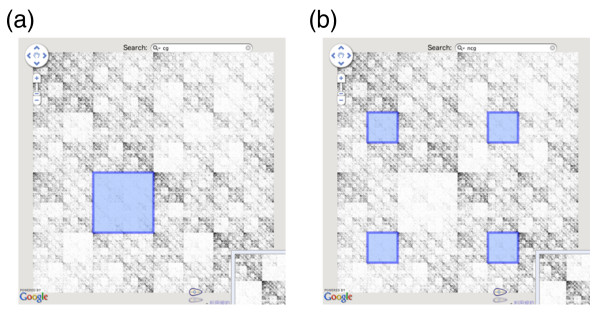
**Web-based interactive and zoomable CGR**. CGR is generated as interactive Google Map image from our server, which allows smooth zooming with mouse scroll wheels, double clicks, or with the controller located in the upper left corner. Oligomers can be searched incrementally from the search box located at the top, either specifically as in (a), or ambiguously as in (b) by using the letter "n" to match all four nucleotides. Search result is immediately highlighted on the map.

A typical interface of interactive and zoomable CGR generated by our server is shown in Figure 3. CGR and *k*-mer table generated by our server is accessible as a Google Map; therefore, users can intuitively and smoothly navigate the large image by panning with mouse dragging, and by zooming in and out with mouse scroll wheels, double clicking, or by using the controller located in the upper left corner. ZUI allow multi-scale observation of large CGR image at varying magnification levels, which is suited to the scale-independent nature of CGR. The coordinate system of CGR and *k*-mer is set to be identical by calculating CGR in reverse sequence order; therefore, *k*-mer table can be used as a guide to locate a certain oligomer and to look up the exact count of the oligomer of interest. Using the overlay feature of Google Maps, CGR and *k*-mer images can be readily switched with the buttons located in the upper right corner. Online examples are available at our website [19] for the genomes of *Escherichia coli *K12 (NC_000913), *Bacillus subtillis *(NC_000964), *Synechococcus sp*. (NC_005070), *Pyrococcus furiosus *(NC_003413), *Mycoplasma genitalium *(NC_000908), *Saccharomyces cerevisiae *chromosome I (NC_001133), *Drosophila melanogaster *chromosome 2R (NT_033778), and *Homo sapiens *chromosome 21 (NT_011512).

In order to fully utilize the CGR as scale-independent Markov transition table, and to be able to quickly locate oligomers in the *k*-mer table, the map can be searched for oligomers of any length from the search box located at the top (Figure 3). Search is incremental, and therefore corresponding position is immediately highlighted within the map upon typing the nucleotide sequence. Oligomers can be searched by specific sequences using only the four nucleotides (Figure 3a), or ambiguously using "n" to represent the all four nucleotides (Figure 3b). 4^n ^regions are highlighted when multiple "n"s are used. With these zooming and interactive searching capabilities, CGR can be a powerful tool in studying the genomic signatures and overrepresented or underrepresented sequence motifs within the genome.

### REST Web service API

The web server is provided as REST web service [20], through G-language REST Server [18]. In principle, our service can be accessed by specifying a URL with the following syntax:

[genome]/[method]/

Here the [genome] is a RefSeq accession number (see here for listing), and [method] is either cgr (for Chaos Game Representation) or kmer_table (for *k*-mer table). For example, for Mycoplasma genitalium genome (RefSeq: NC_000908) is:





Google Map view can be generated by appending "output = gmap" to the above URL, as follows:



In this way, all maps are generated on the fly, and are always up-to-date. Moreover, other web-pages or web-database sites can utilize our service to add CGR and *k*-mer table to their website, by simply referring to our URL. To use our service with user's own sequence, the sequence should be uploaded from  and use the reference ID given by the uploader in place of the accession number. For more details about the service, or the Perl API distributed with the latest G-language GAE package (version 1.8.9 or above) to use the software locally, see the documentations in our website [19].

## List of abbreviations

CGR: Chaos Game Representation; FCGR: Frequency matrices extracted from CGR; G-language GAE: G-language Genome Analysis Environment; IFS: Iterated Function System; REST: Representational State Transfer; ZUI: Zoomable User Interface.

## Competing interests

The authors declare that they have no competing interests.

## Authors' contributions

KA designed and developed the software, and drafted the manuscript. KO participated in the implementation of the software. MT supervised the project. All authors have read and approved the final manuscript.
